# Quo vadis Pantanal? Expected precipitation extremes and drought dynamics from changing sea surface temperature

**DOI:** 10.1371/journal.pone.0227437

**Published:** 2020-01-07

**Authors:** Dirk Thielen, Karl-Ludwig Schuchmann, Paolo Ramoni-Perazzi, Marco Marquez, Wilmer Rojas, Jose Isrrael Quintero, Marinêz Isaac Marques

**Affiliations:** 1 Laboratory of Landscape Ecology and Climate, Venezuelan Institute for Scientific Research (IVIC), Caracas, Venezuela; 2 National Institute for Science and Technology in Wetlands (INAU), Federal University of Mato Grosso, Computational Bioacoustics Research Unit (CO.BRA), Cuiabá, Mato Grosso, Brazil; 3 Postgraduate Program in Zoology, Institute of Biosciences, Federal University of Mato Grosso, Cuiabá, Mato Grosso, Brazil; 4 Zoological Research Museum A. Koenig, Department of Vertebrates, Bonn, Germany; 5 University of Bonn, Faculty of Mathematics and Natural Sciences, Bonn, Germany; 6 Federal University of Lavras, Lavras, Minas Gerais, Brazil; 7 Center of Model Simulation, University of Los Andes, Mérida, Venezuela; 8 Postgraduate Program in Ecology and Biodiversity Conservation, Institute of Biosciences, Federal University of Mato Grosso, Cuiabá, Mato Grosso, Brazil; Indiana State University, UNITED STATES

## Abstract

Climate change poses a critical threat to the Pantanal, the largest wetland in the world. Models indicate an increase in the frequency of extreme precipitation events and extended periods of drought. These changes can amplify consequences for Pantanal’s ecological functioning, which has already experienced intensive human modification of its hydrological system and environmental health. The present study analyzed the spatial and temporal dynamics of rainfall and resulting extremes in the Brazilian area of the Upper Paraguay River Basin (UPRB) along with a co-evaluation of the global Sea Surface Temperature data (SST). The predicted results indicate that wet extreme precipitation events will become more frequent in the highlands, while severe and prolonged droughts triggered by warming SSTs in the Northern Hemisphere (North Atlantic and North Pacific oceans) will affect the Pantanal. The linear relations between precipitation with SST of very specific oceanic regions and even from specific oceanic indexes obtained in the present study significantly improve the forecasting capacity, mainly from a resulting reduction to two months of the lead-time between SST warming to concomitant precipitation impacts, and by explaining 80% of Pantanal´s precipitation variation from major oceanic indexes (e.g., ENSO, PDO, NAO, ATL3). Current SST trends will result in inter- and intra-annual flooding dynamic alterations, drastically affecting the Pantanal ecosystem functioning, with consequences for wildlife diversity and distribution. Regarding the foreseeable global climate and land use change scenarios, the results from the present study provide solid evidence that can be used at different decision-making levels (from local to global) for identifying the most appropriate management practices and effectively achieving sustainability of the anthropic activity occurring in the Pantanal.

## Introduction

The Upper Paraguay River Basin (UPRB) forms part of the upper La Plata River Basin [1] within west-central Brazil. It includes two main regions, the Pantanal, a run-on drainage area occupying the center of the UPRB, and the Highlands (locally referred to as the Planalto), which consists of the surrounding watershed areas at (or above) elevations of 200 m. The Highlands are primarily situated in the eastern and northern parts of the basin and provide approximately 80% of the UPRB outlet stream flow [2].

The Pantanal represents the largest wetland in the world [3–5] and was designated a World Heritage Site by UNESCO in 2000. It is a biodiversity hotspot and plays a major role in climate stability [6]. Here, human activities, which include cattle farming, agriculture, professional and recreational fishing, and contemplative ecotourism [7], rely on ecosystem services. This ecosystem functions as a large reservoir that collects water from the surrounding Highlands during the rainy season and then gradually delivers it to the lower sections of the Paraguay River. This system creates a delay of almost six months before the maximum inflows reach the Paraná River, and this gradual process minimizes downstream flooding [8,9]. The Pantanal itself is subject to a flood pulse that is monomodal, predictable, and low amplitude. The flood level gradient creates a range of major habitats in a complex mosaic of annual and pluri-annual seasonal patterns [10]. The UPRB rainfall shows interannual variability with higher or lower rainwater amounts that have either caused severe floods or pronounced dry seasons. Inter- and intra-annual oscillations between drought and flood serve as primary drivers of the ecological processes and relatively high levels of biodiversity [11,12] found in the region. The combination of hydrological dynamics and geomorphological features specifically help support aquatic, wetland, and terrestrial plant and animal species [13].

Along with expanding agricultural activities and hydroelectric development, climate change poses a major threat to the Pantanal wetland [14,15]. Changes in climatic conditions may cause significant disturbances in ecosystem functioning, mostly by altering the spatial and temporal precipitation dynamics and extreme precipitation events, which in turn affect the fluvial regimes and flooding dynamics. Knowledge of severe floods and droughts is fundamental for wildlife management and nature conservation of the Pantanal [16]. Current predictive climate models indicate a progressive increase in the frequency of extreme events (e.g., extreme rainfalls and extended droughts) [9,17,18]. These events will affect the Pantanal´s ecosystem functioning, amplifying and worsening human modifications of hydrological and environmental conditions in the basin [19].

There is an increasing need to improve the forecasting capabilities of local extreme precipitation events originating from regional to global processes. In this sense, searching for correlations with global climatic phenomena can help resolve the hydrometeorological dynamics of the Pantanal [20]. The analysis of the effects of ocean temperature and atmospheric pressure on land temperature and precipitation patterns has been of increasing concern [21–26]. Previous studies have related Pantanal flooding with the occurrence of two important Sea Surface Temperature (SST) oscillations [27,28]: the El Niño–Southern Oscillation (ENSO) and Pacific Decadal Oscillation (PDO) from the Pacific Ocean; as well as oscillations to SST in the Atlantic Ocean. Proximate locations, such as southern Amazonia, are also affected by the Pacific [7,29], while other close regions, such as the Paraná River Basin, may be affected by the Atlantic oscillations of SST [30].

The exact mechanisms for how SST may influence precipitation at a local level remain uncertain and will not be considered in this study [31]. All previous studies refer to potential connections between local precipitation and SST dynamics at extensive oceanic regions, such as the ones where ENSO, PDO or NAO occur. In the present study, we identified specific oceanic regions in which SST dynamics determine the historical dynamics of the precipitation at the UPRB and its sub-regions. This perspective resulted in a more assertive understanding and modeling of the effects of SST on meteorological events and other hydrological dynamics at the local level [2,32]. Specifically, it considers the variability, frequency, and intensity of any precipitation and/or drought events that are capable of affecting the ecosystem functioning and/or sustainability of human activities that rely on local ecosystem services.

Due to Pantanal’s strong dependence on hydrological factors, changes in temperature and precipitation resulting from climatic change [33,34] may profoundly alter ecosystem functions. Future climate change could affect the hydrological cycle and precipitation patterns in ways that alter the distribution and community composition of tropical biodiversity [35]. Overall, species diversity might decline significantly [36,37]. Using sophisticated spatial analysis tools, this paper interpreted spatial and temporal precipitation dynamics, including extreme precipitation events within the UPRB, and analyzed them in terms of regional climatic parameters (e.g., SST). The results for the two main regions—the Pantanal and the surrounding Highlands—along with their respective sub-regions, indicate there will be major impacts due to current climate change trends. Our results identified spatial and temporal patterns in the SST from specific oceanic regions that appear to influence the local climate conditions in the UPRB.

## Materials and methods

### Study area

**Fig 1:**
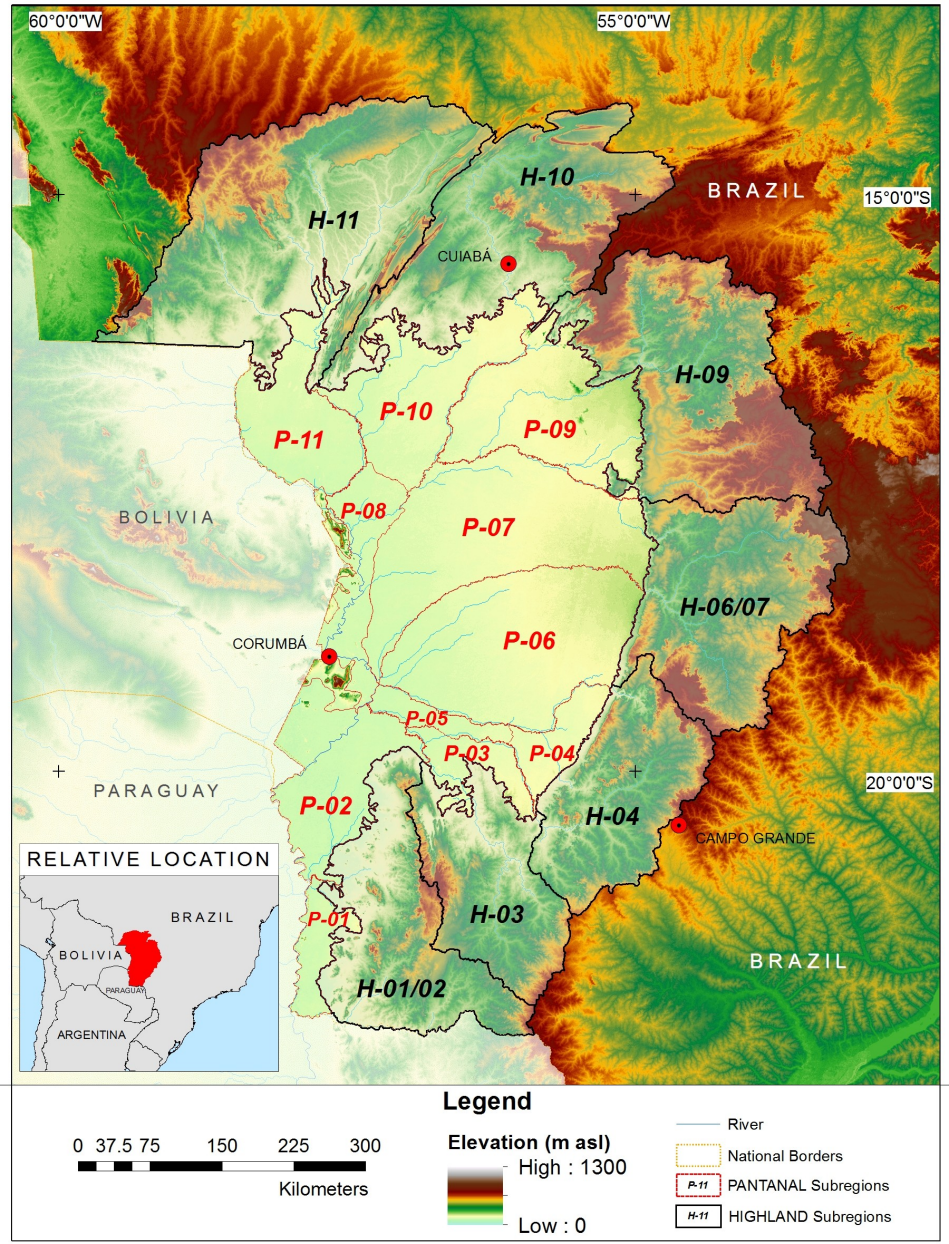
Map of study area. Brazilian area of the Upper Paraguay River Basin (UPRB). Pantanal sub-regions include: Porto Murtinho (P-01), Nabileque (P-02), Miranda (P-03), Aquidauana (P-04), Abobral (P-05), Nhecolândia (P-06), Paiaguás (P-07), Paraguai (P-08), Barão de Melgaço (P-09), Poconé (P-10) and Cáceres (P-11), after Silva and Abdon (1998) [38]. Highland sub-regions (H-01/02, H-03, H-04, H-06/07, H-09, H-10, and H-11), were interpreted as watershed areas (Level 9 of the Pfafstetter Coding System) draining into each Pantanal sub-region.

The study area consisted of regions of the UPRB located within the borders of Brazil (361,338 km^2^). These regions include the lowlands of the Pantanal ecosystem (138,183 km^2^) and the Highlands (223,155 km^2^) surrounding them. According to Silva and Abdon [38], the Pantanal is further divided into 11 sub-regions (Fig 1): Porto Murtinho (P-01; 3,839 km^2^), Nabileque (P-02; 13,281 km^2^), Miranda (P-03; 4,383 km^2^), Aquidauana (P-04; 5,008 km^2^), Abobral (P-05; 2,833 km^2^), Nhecolândia (P-06; 26,921 km^2^), Paiaguás (P-07; 27,082 km^2^), Paraguai (P-08; 8,147 km^2^), Barão de Melgaço (P-09; 18,167 km^2^), Poconé (P-10; 16,066 km^2^), and Cáceres (P-11; 12,456 km^2^). The Highlands were similarly subdivided into seven sub-region-based watersheds (Level 9 of the Pfafstetter coding system) connected to each Pantanal sub-region. These include the H-01/02 (24,461 km^2^) watershed of the Porto Murtinho and Nabileque areas, the H-03 (19,708 km^2^) watershed of the Miranda area, the H-04 (22,371 km^2^) watershed of the Aquidauana area, the H-06/07 (31,530 km^2^) watershed of the Nhecolândia and Paiaguás areas, the H-09 (38,210 km^2^) watershed of the Barão de Melgaço area, the H-10 (35,600 km^2^) watershed of the Poconé area, and the H-11 (51,275 km^2^) watershed of the Cáceres area. Shapefiles for these Pfafstetter Level 9 catchments were accessed at https://www.hydrosheds.org/ [39].

### Data

Precipitation data were obtained from the Climate Hazards Group Infrared Precipitation with Stations (CHIRPS, https://iridl.ldeo.columbia.edu/SOURCES/.UCSB/.CHIRPS/.v2p0/.monthly/.global/). CHIRPS v.2 is a >30 year quasi-global rainfall dataset, spanning 50°S—50°N (all longitudes). From 1981 onwards, CHIRPS v.2 incorporates 0.05° resolution satellite imagery with *in situ* station data to provide rainfall time series with good spatial coverage. The tools associated with the datasets can effectively detect and map precipitation extremes [40].

By means of 23 global-scale precipitation datasets, CHIRPS v.2 was used to perform hydrological modeling for areas of central and eastern Brazil [41]. This study specifically used Jan 1981/Jun 2018 monthly data based on 450 rasters. The ArcGIS software platform (ESRI, Redlands, CA) was applied to process monthly and annual mean rainfall and other basic precipitation parameters.

The monthly mean Sea Surface Temperatures (SST) was provided by the NOAA Extended Reconstructed Sea Surface Temperature Version 5 dataset (ERSSTv5), which is a global monthly SST dataset derived from the International Comprehensive Ocean-Atmosphere Dataset (ICOADS). It is produced on a 2 x 2 degree grid with spatial completeness enhanced using statistical methods (http://iridl.ldeo.columbia.edu/maproom/Global/Ocean_Temp/Monthly_Temp.html).

### Calculation of monthly Standardized Pluviometric Drought Index (SPDI)

Spatiotemporal precipitation patterns were modeled using the Standardized Pluviometric Drought Index (SPDI) [42]. Similar to the Standardized Precipitation Index (SPI) [43], the SPDI is a monthly rainfall index based on cumulative monthly rainfall anomalies. Table 1 shows the categories that result from the application of this index.

**Table 1:** Categories resulting from SPDI estimation.

SPDI values	Category
≥2.00	Extremely humid
1.50–1.99	Very humid
1.00–1.49	Moderately humid
-0.99–0.99	Near normal
-1.00 –-1.49	Moderately dry
-1.50 –-1.99	Very dry
≤ -2.00	Extremely dry

The SPDI is calculated as follows:

The first stage is calculated by Eq (1):

$$APi=Pi-P_{MED}$$ (1)

where *APi* is the monthly precipitation anomaly, *Pi* is the monthly precipitation of month i, and *P_MED_* is the median precipitation for month i within the 1981–2010 time series.

The second stage is calculated by Eq (2):

APAi=∑APiFromi=APnegativetoi=APApositive (2)

where *APAi* represents the accumulated precipitation anomaly for month i.

The third stage is calculated by Eq (3):

$$SPDI=(APAi-\bar{APA})/\sigma APA$$ (3)

where $\bar{APA}$ is the average value of the accumulated precipitation anomalies of all the months in the series, and *σAPA* is the standard deviation of the accumulated precipitation anomalies of all the months in the series.

ArcGIS allowed us to generate SPDI estimates based on the 450 CHIRPS v.2 rasters and 0.05° resolution images. The procedures also provided monthly zonal estimates and estimates based on different space and/or time criteria. The monthly SPDI values reported above were estimated based on normal precipitation for each of the Pantanal and Highland sub-regions as estimated from a 1981–2010 study period (Fig 1). Pearson correlation coefficients were used to estimate the covariance (α = 0.01) between monthly precipitation and/or SPDI values for the different areas. Statistically significant differences were evaluated using a two-tailed, paired t-test.

### Correlation of UPRB precipitation with Sea Surface Temperature (SST)

For the 1981–2018 analysis period, GIS analytical tools were used to investigate correlations between mean monthly SST and mean monthly precipitation in the UPRB. Analysis assumed that the observed SST preceded precipitation by lead times of 0 to 6 months. The Pearson correlation coefficient was used to estimate the degree of explained variance (α = 0.01) between the monthly SST and precipitation. A paired, two-tailed t-test was used to assess the significance of statistical differences. Confidence intervals of 99% were used to identify correlation coefficients (r) ranges. Correlation rasters and associated r values were used to identify the spatial area of oceanic regions with the highest correlation coefficients for observed precipitation (1981–2018) over a range of different lead times. Mann-Kendall tests were applied to determine the significance of the time series trends in the monthly SSTs for each oceanic zone.

## Results

### Precipitation

**Fig 2:**
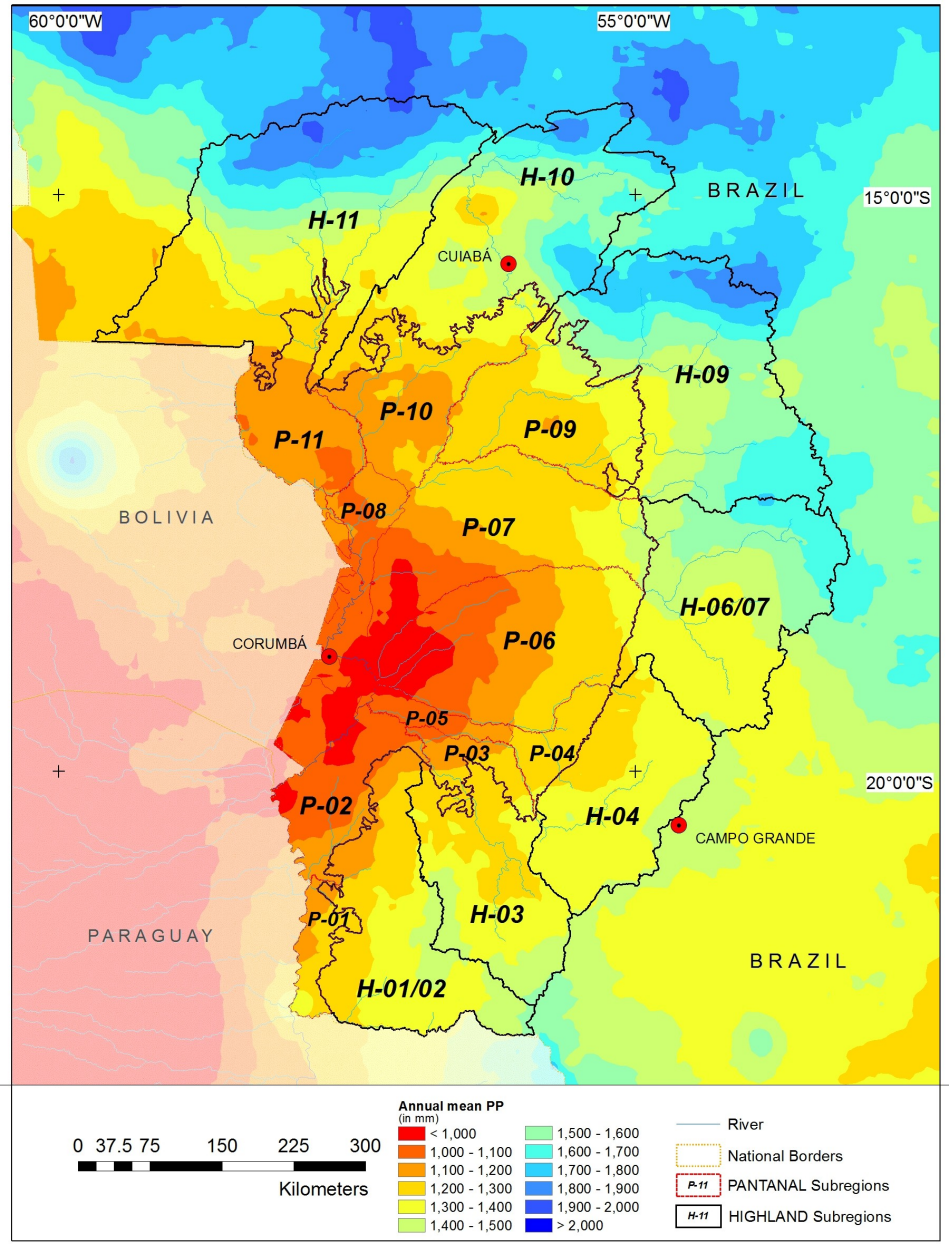
Annual mean precipitation within the Upper Paraguay River Basin (UPRB). Sub-regions are referred to in Fig 1.

As for the UPRB, the 1981–2010 series of annual mean precipitation was 1346 mm. For the same period, the Highlands experienced significantly wetter conditions (1454 mm) than those of the Pantanal (1176 mm; P < 0.001). Datasets revealed an annual north-south precipitation gradient ranging from 1312 to 1572 mm (Fig 2). No significant differences were detected among any of the Highland sub-regions (paired t-test; P > 0.05). The Pantanal showed significant spatial variation in its annual precipitation. The Pantanal sub-regions of Nabileque, Abobral, and Paraguai experienced precipitation amounts of 1044, 1059, and 1065 mm year^-1^, respectively. These areas were significantly drier (P < 0.05) than the sub-regions of Nhecolândia, Cáceres, Paiaguás, and Miranda (1144, 1156, 1188, and 1205 mm year^-1^, respectively). The former sub-regions were also significantly drier (P < 0.001) than the wettest sub-regions of the Pantanal, e.g., Poconé, Porto Murtinho, Aquidauana, and Barão de Melgaço receiving 1231, 1232, 1256, and 1295 mm year^-1^, respectively.

**Fig 3:**
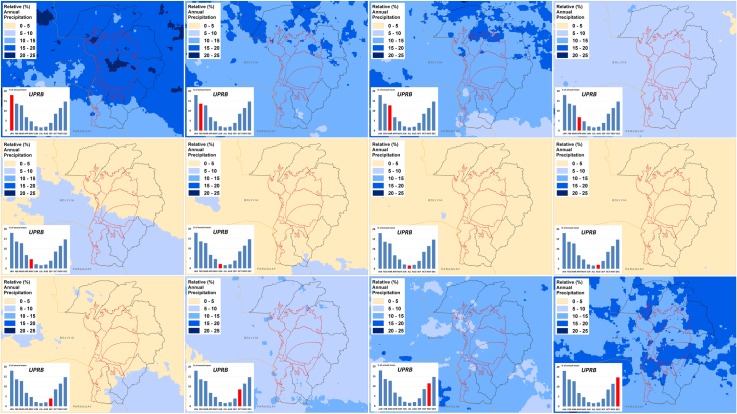
Relative (%) monthly precipitation distribution for the Upper Paraguay River Basin (UPRB).

Data analysis of the relative (%) annual precipitation distribution within the UPRB detected a significant (P < 0.001) positive correlation (r > 0.98) between sub-regions, especially in areas connected to the Highlands and/or the Pantanal. This pattern likely arises from the generalized unimodal precipitation distribution, wherein approximately 80% of the total annual precipitation occurs from October to March (Fig 3). The analysis of monthly data showed no significant difference (P > 0.05) in the distribution of precipitation between the Highlands and/or Pantanal sub-regions for the 1981–2010 time series.

**Fig 4:**
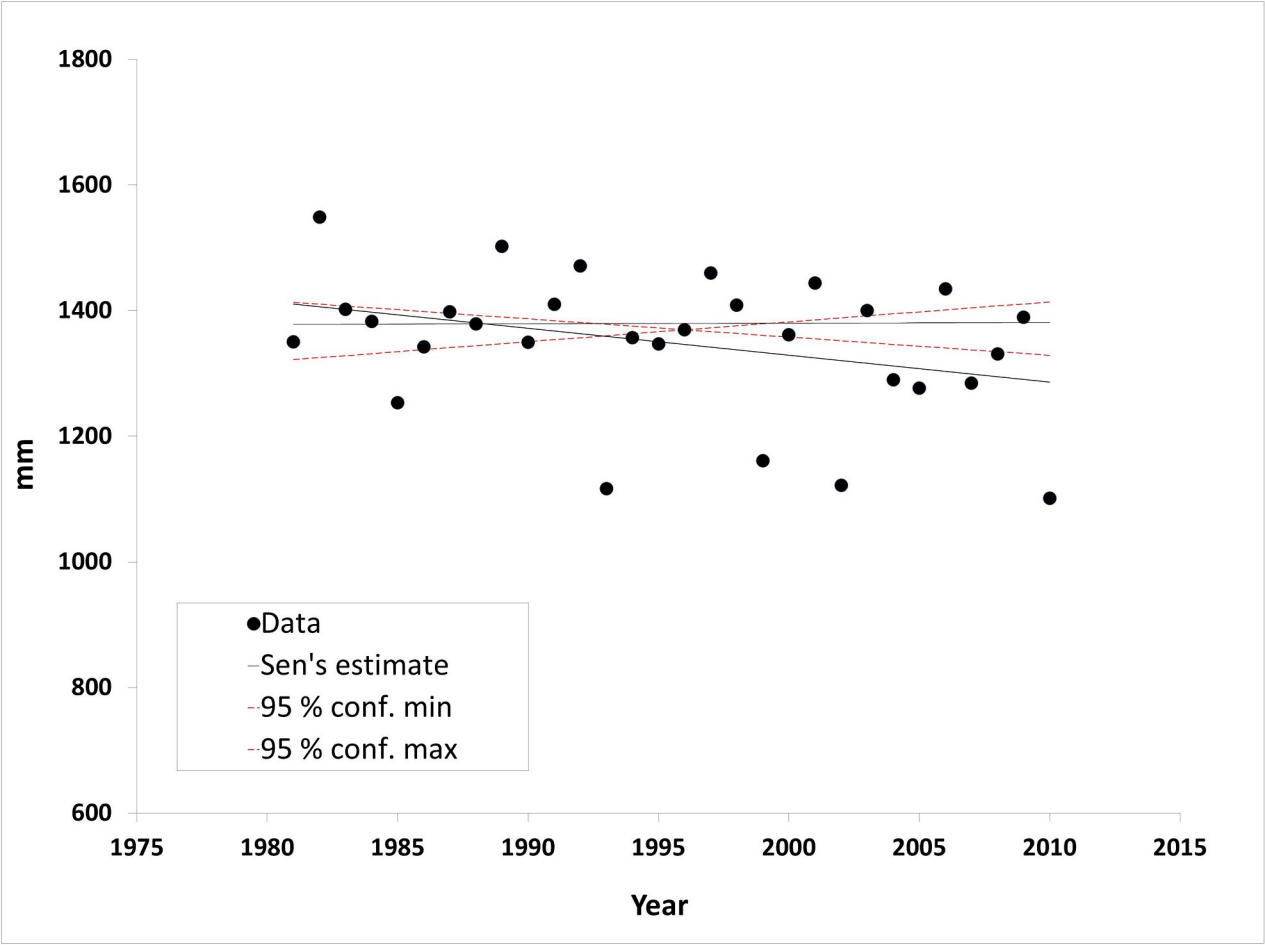
Inter-annual precipitation dynamics (1981–2017) for the Upper Paraguay River Basin (UPRB).

The annual precipitation from 1981 to 2017 tended to vary more in the Pantanal than in the Highlands (±121 vs. ±109 mm year^-1^, respectively). The southernmost sub-regions of the Pantanal experienced the greatest variability: ±180 mm for Porto Murtinho, ±177 mm for Aquidauana, ±164 mm for Abobral, ±162 mm for Miranda, and ±148 mm for Nabileque. Within the Highlands sub-regions, the highest degree of variation also occurred in the southerly regions: ±171 mm for H-04, ±165 mm for H-01/02, and ±160 mm for H-03. Fig 4 illustrates the absence of a significant annual precipitation trend in the 1981–2017 time series data for the UPRB (Mann-Kendall test, P > 0.05). Data from the Highlands, the Pantanal, and the individual sub-regions similarly showed no significant trends (neither increase nor decrease) in annual precipitation (Table 2).

**Table 2:** Mann-Kendall slope and trend significance test for the time series 1981–2017 for the Upper Paraguay River Basin (UPRB), the Highlands, and the Pantanal areas as well as their different sub-regions (see Fig 1).

	Test Z	Signific.	Q	Q_min99_	Q_max99_	B	B_min99_	B_max99_
**Highlands**	0.48391	NS	1.06386	-4.03853	5.2212	1437.9740	1662.6453	1246.4184
*H-01/02*	0.40544	NS	1.56956	-7.15099	9.4380	1271.1179	1658.3511	924.9062
*H-03*	0.79781	NS	2.69054	-6.25389	11.8387	1272.8659	1682.6248	848.1280
*H-04*	0.98091	NS	3.03922	-5.02400	10.5800	1249.6635	1621.0632	877.1471
*H-06/07*	0.43160	NS	1.35461	-5.67938	7.0929	1401.6356	1744.4750	1135.4599
*H-09*	0.40544	NS	0.91576	-4.35379	6.2211	1555.2508	1814.9227	1304.1071
*H-10*	0.71933	NS	1.53739	-3.72842	6.3054	1434.8234	1680.4287	1168.8582
*H-11*	-0.90244	NS	-2.24385	-8.07633	3.5285	1620.5682	1907.6034	1347.4866
**Pantanal**	-0.82397	NS	-1.41484	-5.85063	3.3367	1253.5647	1474.6748	1044.1982
Porto Murtinho (***P-01***)	-0.64086	NS	-2.29374	-10.6062	7.56773	1351.5197	1726.0165	909.6009
Nabileque (***P-02***)	0.03923	NS	0.16514	-5.56805	6.79478	1073.6769	1357.7034	736.3475
Miranda (***P-03***)	1.26865	NS	2.35717	-3.23933	9.17391	1146.1971	1410.1595	806.6391
Aquidauana (***P-04***)	0.71933	NS	2.00247	-5.24114	8.79745	1210.4004	1539.7506	878.1254
Abobral (***P-05***)	0.03923	NS	0.16095	-6.63037	7.02612	1096.7412	1408.4170	739.3102
Nhecolândia (***P-06***)	-0.14386	NS	-0.26106	-5.24051	5.64936	1197.8901	1428.9086	924.8686
Paiaguas (***P-07***)	-1.39944	NS	-2.25553	-7.74219	2.72160	1303.8043	1555.5458	1071.1621
Paraguai (***P-08***)	-1.45028	NS	-1.54228	-4.13419	1.15229	1143.3730	1229.8437	1035.3777
Barão de Melgaço (***P-09***)	-0.51007	NS	-1.64431	-7.29734	5.27726	1370.1871	1640.4848	1076.0669
Poconé (***P-10***)	-0.64086	NS	-1.17561	-6.90496	4.34565	1297.1701	1556.9255	1036.1247
Cáceres (***P-11***)	-1.39944	NS	-3.29740	-7.96864	2.89571	1327.9828	1540.3516	1011.3098
**UPRB**	0.03923	NS	0.10499	-4.27405	4.10772	1374.9776	1571.0541	1186.4188

### Monthly Standardized Pluviometric Drought Index (SPDI)

**Fig 5:**
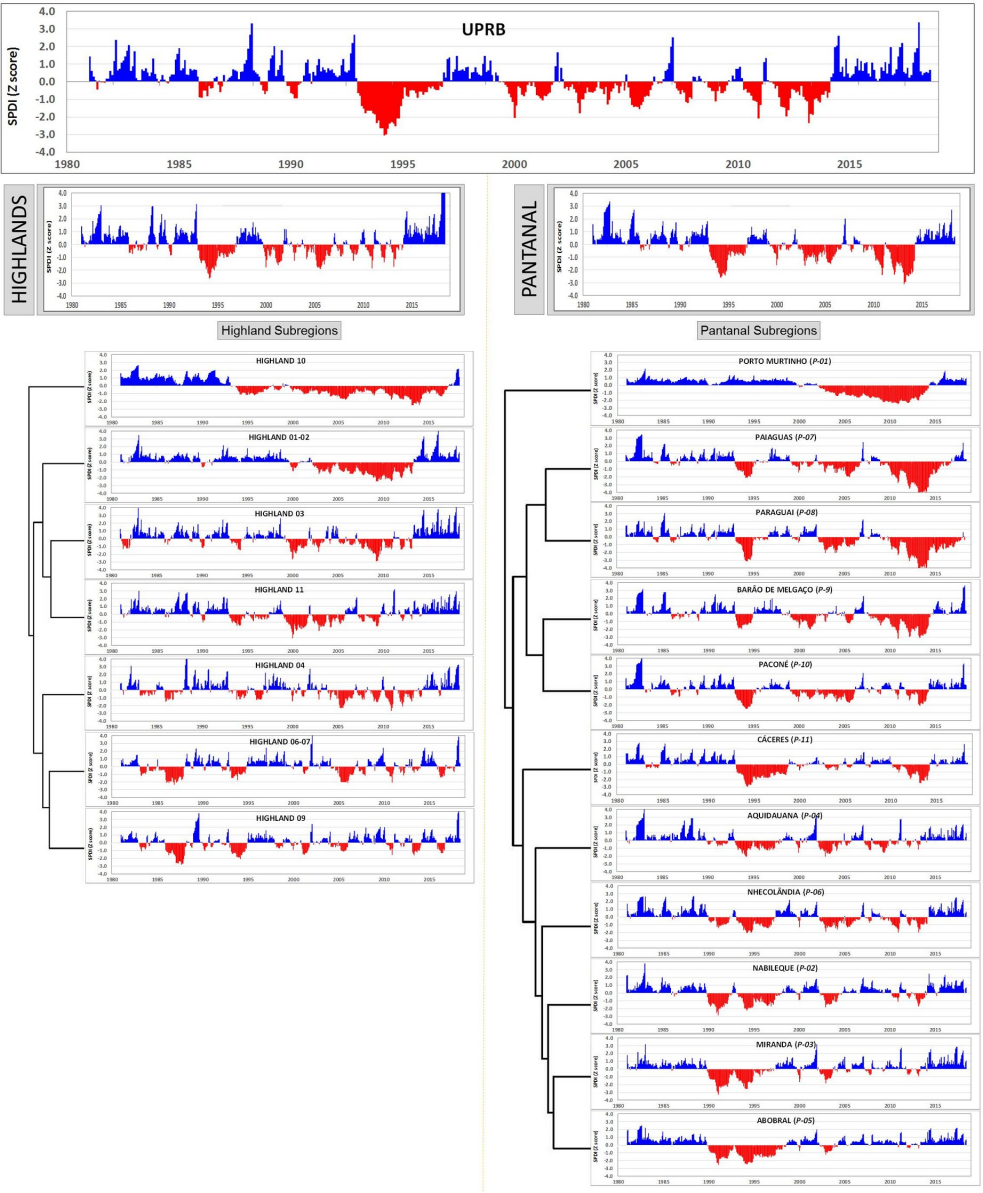
Temporal dynamics in the Standardized Pluviometric Drought Index (SPDI) for the Upper Paraguay River Basin (UPRB). Here, sub-regions are clustered using correlation distance and complete linkage estimated using the statistical tool ClustVis [44]. For each graph, precipitation conditions estimated as SPDI were interpreted using the classification system from Table 1: where ≤ -2.0 is extremely dry, -1.99 to -1.5 is very dry, -1.49 to -1.0 is moderately dry, -0.99 to 0.99 near normal, 1.0 to 1.49 is moderately wet, 1.5 to 1.99 is very wet, and > 2.0 is extremely wet.

Within the UPRB (Fig 5), 72.2% of the 450 months that comprised the January 1981 to June 2018 study period exhibited near-normal SPDI values [43] (from -0.99 to 0.99). Some (15.1%) of the months qualified as being wet (SPDI ≥ 1.0), 7.6% were moderately wet (1.0 to 1.49), 3.3% were very wet (1.5 to 1.99), and 4.2% were extremely wet (SPDI ≥ 2.0). Likewise, 12.7% of the months were dry (SPDI ≤ -1), 6.2% were moderately dry (-1.0 to -1.49), 2.9% were very dry (-1.5 to -1.99), and 3.6% were extremely dry (SPDI ≤ -2.0). Wet conditions in the UPRB occurred primarily over short durations, with 90.6% of wet events lasting only one to two months (SPDI mean intensity of 1.35). Other wet events lasted from three (n = 6, mean intensity 2.00) to five consecutive months (n = 3, mean intensity 2.12). The 1981–2018 study period had a maximum SPDI value of 4.01, which was reached at the end of a five-month wet spell. On the other hand, dry conditions within the UPRB were less frequent but had more persistent pulses (Fig 5). There were four pulses with a two-month duration (mean intensity -1.23), one three-month pulse (intensity -1.51), three five-month pulses (intensity -1.52), one seven-month pulse (intensity -1.34), and a protracted drought of 22 months (from April 1993 to December 1994, with a mean intensity of -2.16). The historical minimum SPDI value of -3.10 occurred in the middle of this major drought.

Fig 5 also illustrates that the spatiotemporal variation in precipitation introduces considerable overall variation to the study area. For example, the monthly SPDI for the Highlands strongly contrasted with that of the Pantanal (P < 0.001). Although there was no significant difference (P = 0.6167) in the frequency of normal precipitation (SPDI from -0.99 to 0.99) between the Highlands and the Pantanal (71.6% and 70.7%, respectively), these regions varied in terms of the frequency of their wet extreme events (15.8% in the Pantanal vs. 11.6% in the Highlands). In contrast, dry events occurred more frequently in the Pantanal (17.8%) than in the Highlands (12.8%). The Highlands experienced a maximum SPDI of 5.09, while the Pantanal reached a maximum SPDI of 3.36. Furthermore, the Pantanal experienced a historically (1981–2017) low SPDI value of -3.12 (vs. -2.65 for the Highlands).

Within the Highlands, 50.8% of months with abnormal SPDI values (i.e., < -1.0, > 1.0) occurred during relatively short wet or dry pulses (< 3 months). Of the remaining 49.2% of the months, 22.5% showed an extreme wet event that lasted approximately 8 months, while 75.4% of the protracted dry pulses lasted from 8 to 23 months. In the Pantanal region, 70.4% of the months with abnormal SPDI values (i.e., < -1.0, > 1.0) occurred as protracted pulses (> 4 months) and dry events (92.5%). In the Pantanal, 63.5% of the months observed as abnormally wet did not occur as protracted events. Temporal dynamics were also evident among the different sub-regions from both the Highlands and the Pantanal. Cluster analysis (Fig 5) identified three main groups among the Highlands sub-regions.

**Fig 6:**
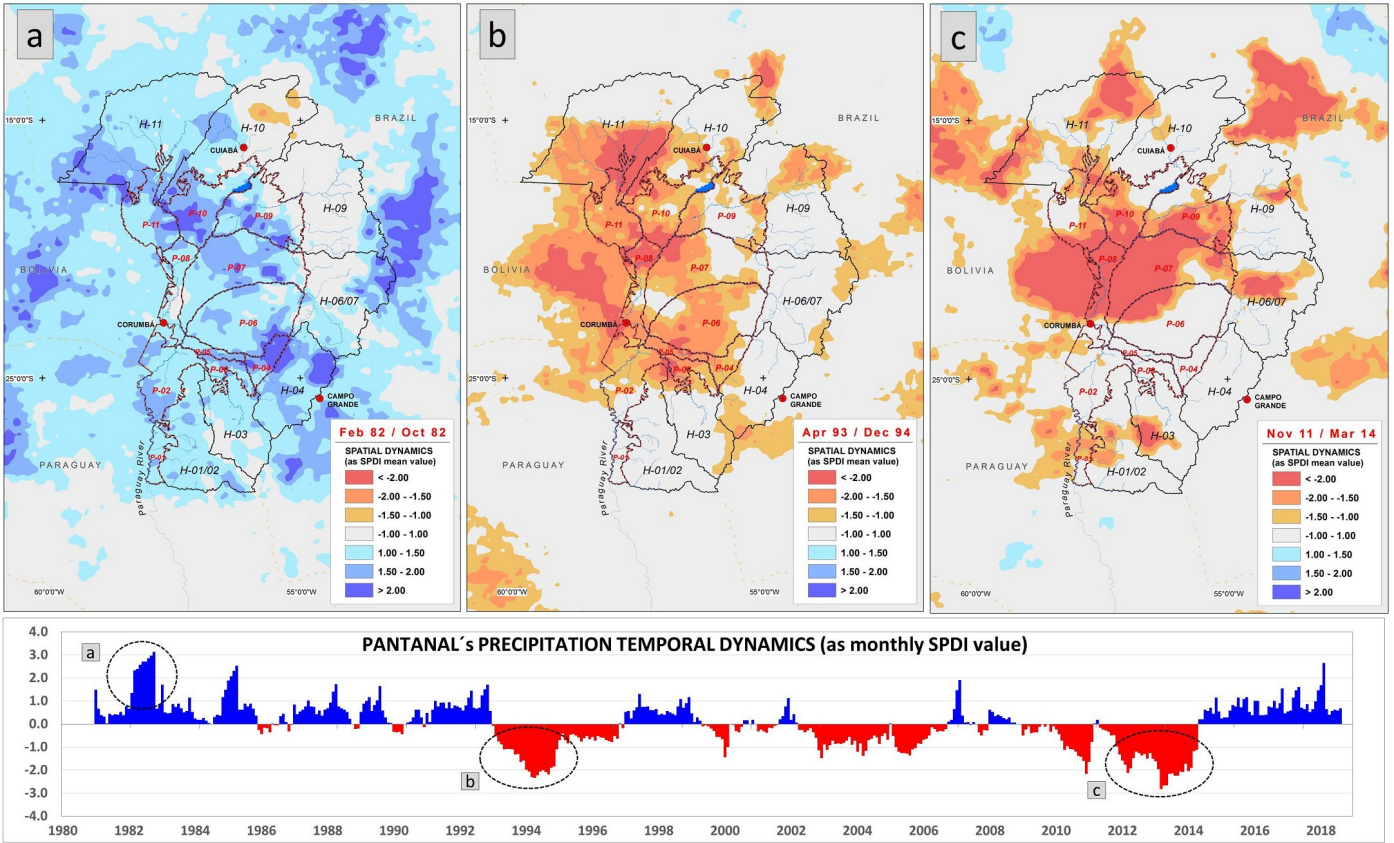
Historic extreme precipitation events affecting the study area. Panel (a) shows an extremely wet pulse (intensity 2.74±0.57, duration 9 consecutive months, Feb/Oct 1982), (b) a very dry pulse (intensity -1.87±0.53, duration 21 months, Apr 1993/Dec 1994), and (c) a very dry pulse (intensity -1.98±0.54, duration 30 months, Nov 2011/Mar 2014). Precipitation conditions described by classification system from Table 1.

The first of these clusters consisted of the two southernmost sub-regions (H-01/02, H-03) along with the northernmost one (H-11). For at least the first 18 years of the 1981–2018 study period, the first cluster exhibited similar SPDI values, indicating normal to moderately wet conditions (SPDI from 1.0 to 1.49); however, there were as many as six wet pulses with SPDI values **≥** 2.0 (extremely wet). These wet pulses lasted only 2–8 months. Fig 6A shows the spatial distribution of one such wet pulse. From 1999 to 2012, the SPDI values indicate much drier conditions, from moderately dry (SPDI from -1.0 to -1.49) to extremely dry (≤ -2.0), expressed as pulses lasting for several years. Over the last five years of the study period (2013–2017), the three sub-regions experienced normal to moderately wet conditions that were interspersed with frequent extreme precipitation events lasting from 2 to 4 months.

The second major group identified by cluster analysis included the geographically intermediate Highlands sub-regions H-04, H-06/07, and H-09 (Fig 5). This group had SPDI values with greater temporal variation that was introduced by several major dry periods interspersed with wet pulses. One dry period lasted for two years (Jan 1986/Dec 1987), affecting mainly H-06/07 and H-09, which exhibited drought intensities of -1.76 and -1.88, respectively. Another drought, qualified as moderate (-1.49), lasted 19 months (Apr 1993/Dec 1994), affecting mainly H-09 and, to a lesser extent, the higher elevation areas of H-06/07 and H-04 (Fig 6B).

A third and final cluster represented the distinct temporal SPDI dynamics of H-10 (Fig 5). This northernmost sub-region showed a 12-year continuum (Jan 1981/Dec 1992) of positive SPDI values that were interspersed with wetter pulses of varying intensity. This stretch was followed by a dry period of 24 years (Jan 1993/Dec 2016) with varying pulses of negative SPDI values. For the last 15 months of the study period (Jan 2017/Jun 2018), this sub-region exhibited positive SPDI values with a very wet pulse (+1.85) beginning in Jan 2018. Extreme precipitation events, such as an extremely wet pulse in Feb 1982/Oct 1983 (Fig 6A) and the two very dry pulses in Apr 1993/Dec 1994 and Nov 2011/Mar 2014 (Fig 6B and 6C, respectively), also distinguished this Highlands sub-region from others.

As with the Highlands, cluster analysis of the SPDI values for sub-regions of the Pantanal identified three distinct clusters. The first cluster, which was composed of Abobral, Miranda, Nabileque, Nhecolândia, Aquidauana, and Cáceres, experienced a distinctive wet phase in Jan 1981/Dec 1989. This phase included several wet pulses of varying intensities and durations. The most extreme occurred in Feb 1982/Oct 1982 (Fig 6A). With the exception of Miranda and Nabileque, which exhibited very wet SPDI values (1.64 and 1.91, respectively), all other sub-regions from these clusters exhibited extremely wet SPDI values (≥ 2.0). From Jan 1990 to Aug 1998, the six sub-regions from this cluster entered a prolonged dry phase, which was characterized by two very distinct and extreme events. The first occurred from Jan 1990 to Apr 1992 and generated drought conditions from very dry to extremely dry in the Abobral, Miranda, and Nabileque sub-regions; however, the drought conditions were less intense in the remaining three sub-regions (Fig 5). The second dry pulse, indicated in Fig 6B, occurred in Apr 1993/Dec 1994, during which extreme drought conditions occurred throughout the Pantanal ecosystem. Drought persisted in these sub-regions, albeit with relatively high SPDI values, until the end of 1997. For the next 17 years, the precipitation varied sufficiently to generate short pulses of varying intensities and/or to generate alternating dry and wet conditions. From the beginning of 2014 to the present, the SPDI values within the six Pantanal sub-regions indicate generally wet conditions (SPDI > 1.0), and these conditions have been interspersed with abrupt, extremely wet events, such as the one that occurred during the 2014/15 rainy season (SPDI ≥ 2.0).

The second Pantanal cluster consisted of the Poconé, Barão de Melgaço, Paraguai, and Paiaguás sub-regions. From Jan 1981 to Dec 1989, this cluster exhibited SPDI dynamics that indicated generally wet conditions. In contrast to the cluster described above, this second cluster experienced brief and severe wet events as well as short, low intensity dry events. The cluster also experienced an extreme wet event in Feb 1982/Oct 1982 (Figs 5 and 6A). A second extreme wet event, lasting six months and with an SPDI value ≥ 2.0, occurred during the Nov 1984 to May 1985 rainy season. The prevailing wet conditions of the first nine years ended with the extreme dry event in Apr 1993/Dec 1994 (Figs 5 and 6B). The dry pulse was least extreme in Barão de Melgaço (SPDI -1.48) and most extreme in Paraguai (SPDI -2.39). From here on, the SPDI was highly variable among the sub-regions of this cluster. During this time and until Nov 2011, dry spells of varying durations occurred at different times in the four sub-regions of this cluster. A protracted and severe drought affected Paiaguás (SPDI -3.38), Paraguai (SPDI -3.09), and Barão de Melgaço (SPDI -2.28) and to a lesser extent, Poconé (SPDI -1.67) (Figs 5 and 6C). Except for Paraguai, these sub-regions experienced wet conditions from mid-2014 to the present along with an extreme wet event (SPDI 2.32) that occurred during the last rainy season of the 1981–2018 study period.

The SPDI dynamics of the southernmost sub-region of Porto Murtinho were sufficiently unique and qualified as a separate cluster. Precipitation dynamics created wet conditions in Jan 1981/Dec 2003. In addition to an extreme wet event in Feb 1982/Oct 1982 (Figs 5 and 6A), when the mean SPDI for this sub-region reached a value of 1.6, the precipitation rarely generated an SPDI in excess of 1.20 for more than a month. In Jan 2004/Sep 2013, the SPDI values ranged from very dry (-1.5, -2.0) to extremely dry (≤ -2.0), indicating a sustained drought. As with most of the Pantanal sub-regions, the SPDI values for Porto Murtinho from the beginning of 2014 to the present indicated wet conditions.

Fig 6A shows an extreme wet pulse in Feb 1982/Oct 1982 that primarily affected the Pantanal lowlands rather than the adjacent Highlands (90.5% vs. 63.7% of their respective areas). This event caused an SPDI > 1.56 for 40.1% of the Pantanal area vs. 20.6% of the Highlands. The very dry events of Apr 1993/Dec 1994 and Nov 2011/Mar 2014 affected a larger area of the Pantanal (~46% of the area, with an SPDI < -1.5 for each event) than that of the Highlands (~16% of the area, with an SPDI < -1.5 for each event).

### Correlation of precipitation with Sea Surface Temperature (SST)

**Fig 7:**
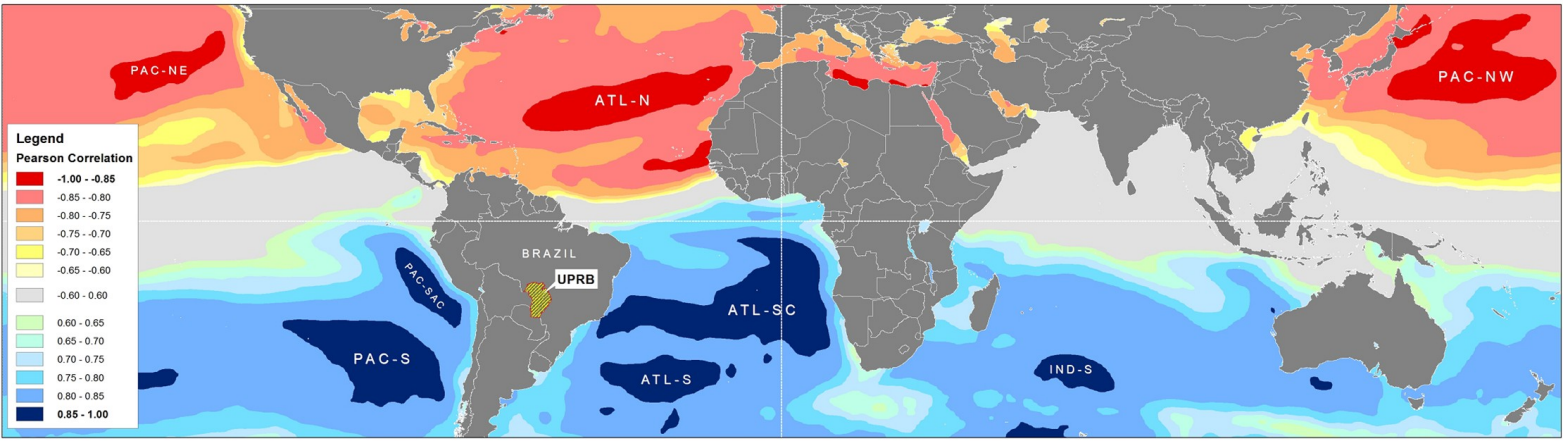
Sea Surface Temperatures (SST) correlation with monthly mean precipitation for the Upper Paraguay River Basin (UPRB). Highlighted oceanic regions show the highest correlation (Pearson) for a two month lead time (i.e., SSTs leading precipitation). The oceanic regions are as follows: ATL-N–Atlantic North, ATL-SC–Atlantic South Central, ATL-S–Atlantic South, IND-S–Indian South, PAC-NE–Pacific North East, PAC-NW–Pacific North West, PAC-SAC–Pacific South American Coast, and PAC-S–Pacific South.

Fig 7 illustrates the oceanic regions with a historical SST correlated (Pearson) with UPRB monthly precipitation. The SST data came from three Atlantic Ocean regions (ATL-N–Atlantic North, ATL-SC–Atlantic South Central, and ATL-S–Atlantic South), four Pacific Ocean regions (PAC-NE–Pacific Northeast, PAC-NW–Pacific Northwest, PAC-SAC–Pacific South American Coast, and PAC-S–Pacific South), and one Indian Ocean region (IND-S–Indian South). As shown in Table 3, most of these oceanic regions indicate a significant (P < 0.01) correlation of the SST values measured two months prior to the precipitation data. In the case of IND-S, a one-month lead time produced the highest correlation coefficients. Generally, the Northern Hemisphere oceanic regions (ATL-N, PACNE, and PAC-NW) were negatively correlated (r < -0.87) with UPRB precipitation, while the Southern Hemisphere regions (ATL-SC, ATL-S, PAC-SAC, PAC-S, and IND-S) had positive correlation coefficients (r > 0.87).

**Table 3:** Temporal correlation (Pearson) for different lead times between Upper Paraguay River Basin (UPRB) monthly mean precipitation and Sea Surface Temperature (SST) for several oceanic regions.

Oceanic Region*	SST lead PP	Correlation coefficient (*r*)	P-value from equation:	Significance level	*r* must exceed: ±	Standard error of the estimate	*r*-squared	Confidence level	*r* range	
PAC-NW	6	0.5729	8.75109E-41	0.01	0.1210	2.9423	32.8%	0.99	0.4854	0.6491
	5	0.1483	0.001566803	0.01	0.1210	3.5501	2.2%	0.99	0.0276	0.2648
	4	-0.3159	6.22412E-12	0.01	0.1210	3.4060	10.0%	0.99	-0.4210	-0.2024
	3	-0.6927	7.34923E-66	0.01	0.1210	2.5891	48.0%	0.99	-0.7509	-0.6239
	**2**	**-0.8832**	**4.8966E-150**	**0.01**	**0.1210**	**1.6839**	**78.0%**	**0.99**	**-0.9073**	**-0.8533**
	1	-0.8396	2.3361E-121	0.01	0.1210	1.9498	70.5%	0.99	-0.8721	-0.7998
	0	-0.5763	2.36906E-41	0.01	0.1210	2.9338	33.2%	0.99	-0.6520	-0.4892
ATL-N	6	0.5614	6.71244E-39	0.01	0.1210	1.5711	31.5%	0.99	0.4723	0.6391
	5	0.1384	0.003184196	0.01	0.1210	1.8803	1.9%	0.99	0.0175	0.2554
	4	-0.3272	9.69158E-13	0.01	0.1210	1.7940	10.7%	0.99	-0.4313	-0.2145
	3	-0.6969	5.83481E-67	0.01	0.1210	1.3616	48.6%	0.99	-0.7544	-0.6288
	**2**	**-0.8824**	**1.8183E-149**	**0.01**	**0.1210**	**0.8932**	**77.9%**	**0.99**	**-0.9067**	**-0.8524**
	1	-0.8303	2.5401E-116	0.01	0.1210	1.0581	68.9%	0.99	-0.8645	-0.7884
	0	-0.5525	1.7224E-37	0.01	0.1210	1.5824	30.5%	0.99	-0.6315	-0.4623
PAC-NE	6	0.5491	5.78092E-37	0.01	0.1210	2.1512	30.2%	0.99	0.4584	0.6285
	5	0.1307	0.005371116	0.01	0.1210	2.5520	1.7%	0.99	0.0097	0.2480
	4	-0.3302	5.86676E-13	0.01	0.1210	2.4297	10.9%	0.99	-0.4340	-0.2177
	3	-0.6960	1.02072E-66	0.01	0.1210	1.8484	48.4%	0.99	-0.7536	-0.6277
	**2**	**-0.8771**	**1.9225E-145**	**0.01**	**0.1210**	**1.2362**	**76.9%**	**0.99**	**-0.9024**	**-0.8459**
	1	-0.8217	5.9945E-112	0.01	0.1210	1.4670	67.5%	0.99	-0.8575	-0.7780
	0	-0.5477	9.52888E-37	0.01	0.1210	2.1536	30.0%	0.99	-0.6273	-0.4568
ATL-SC	6	-0.4995	6.71269E-30	0.01	0.1210	1.5416	25.0%	0.99	-0.5853	-0.4027
	5	-0.0663	0.159618844	0.01	0.1210	1.7756	0.4%	0.99	-0.1860	0.0554
	4	0.3909	6.00686E-18	0.01	0.1210	1.6379	15.3%	0.99	0.2830	0.4889
	3	0.7437	1.00646E-80	0.01	0.1210	1.1896	55.3%	0.99	0.6841	0.7934
	**2**	**0.8961**	**6.982E-161**	**0.01**	**0.1210**	**0.7897**	**80.3%**	**0.99**	**0.8694**	**0.9177**
	1	0.8067	7.7852E-105	0.01	0.1210	1.0517	65.1%	0.99	0.7597	0.8452
	0	0.4986	8.74829E-30	0.01	0.1210	1.5425	24.9%	0.99	0.4017	0.5845
PAC-SAC	6	-0.4457	1.90567E-23	0.01	0.1210	2.0732	19.9%	0.99	-0.5379	-0.3430
	5	-0.0141	0.764971423	0.01	0.1210	2.3158	0.0%	0.99	-0.1351	0.1073
	4	0.4296	1.00293E-21	0.01	0.1210	2.0914	18.5%	0.99	0.3253	0.5236
	3	0.7580	1.46341E-85	0.01	0.1210	1.5105	57.5%	0.99	0.7013	0.8053
	**2**	**0.8852**	**1.1881E-151**	**0.01**	**0.1210**	**1.0775**	**78.4%**	**0.99**	**0.8558**	**0.9089**
	1	0.7751	8.91981E-92	0.01	0.1210	1.4633	60.1%	0.99	0.7217	0.8194
	0	0.4585	7.04038E-25	0.01	0.1210	2.0582	21.0%	0.99	0.3571	0.5492
PAC-S	6	-0.6251	2.30212E-50	0.01	0.1210	1.5781	39.1%	0.99	-0.6937	-0.5452
	5	-0.2200	2.33802E-06	0.01	0.1210	1.9722	4.8%	0.99	-0.3323	-0.1014
	4	0.2456	1.23195E-07	0.01	0.1210	1.9598	6.0%	0.99	0.1282	0.3562
	3	0.6515	6.03684E-56	0.01	0.1210	1.5338	42.4%	0.99	0.5757	0.7162
	**2**	**0.8814**	**1.0472E-148**	**0.01**	**0.1210**	**0.9548**	**77.7%**	**0.99**	**0.8512**	**0.9059**
	1	0.8744	1.9141E-143	0.01	0.1210	0.9809	76.5%	0.99	0.8425	0.9002
	0	0.6311	1.36871E-51	0.01	0.1210	1.5683	39.8%	0.99	0.5521	0.6989
ATL-S	6	-0.6024	5.64135E-46	0.01	0.1210	1.6344	36.3%	0.99	-0.6743	-0.5190
	5	-0.2064	9.69674E-06	0.01	0.1210	2.0035	4.3%	0.99	-0.3196	-0.0874
	4	0.2516	5.89958E-08	0.01	0.1210	1.9817	6.3%	0.99	0.1345	0.3618
	3	0.6471	5.65093E-55	0.01	0.1210	1.5611	41.9%	0.99	0.5706	0.7124
	**2**	**0.8716**	**2.0873E-141**	**0.01**	**0.1210**	**1.0038**	**76.0%**	**0.99**	**0.8390**	**0.8979**
	1	0.8592	4.5873E-133	0.01	0.1210	1.0476	73.8%	0.99	0.8238	0.8880
	0	0.6164	1.21997E-48	0.01	0.1210	1.6124	38.0%	0.99	0.5351	0.6863
IND-S	6	-0.6395	2.44172E-53	0.01	0.1210	1.6710	40.9%	0.99	-0.7060	-0.5618
	5	-0.2464	1.11501E-07	0.01	0.1210	2.1064	6.1%	0.99	-0.3570	-0.1291
	4	0.2132	4.83599E-06	0.01	0.1210	2.1235	4.5%	0.99	0.0944	0.3260
	3	0.6236	4.55278E-50	0.01	0.1210	1.6991	38.9%	0.99	0.5434	0.6925
	2	0.8671	2.8992E-138	0.01	0.1210	1.0828	75.2%	0.99	0.8334	0.8943
	**1**	**0.8755**	**2.9429E-144**	**0.01**	**0.1210**	**1.0501**	**76.7%**	**0.99**	**0.8439**	**0.9011**
	0	0.6460	9.78532E-55	0.01	0.1210	1.6591	41.7%	0.99	0.5693	0.7115

* PAC-NW–Pacific North West, ATL-N–Atlantic North, PAC-NE–Pacific North East, ATL-SC–Atlantic South Central, PAC-SAC–Pacific South American Coast, PAC-S–Pacific South, ATL-S–Atlantic South, and IND-S–Indian South. Fig 7 shows locations of oceanic regions.

**Fig 8:**
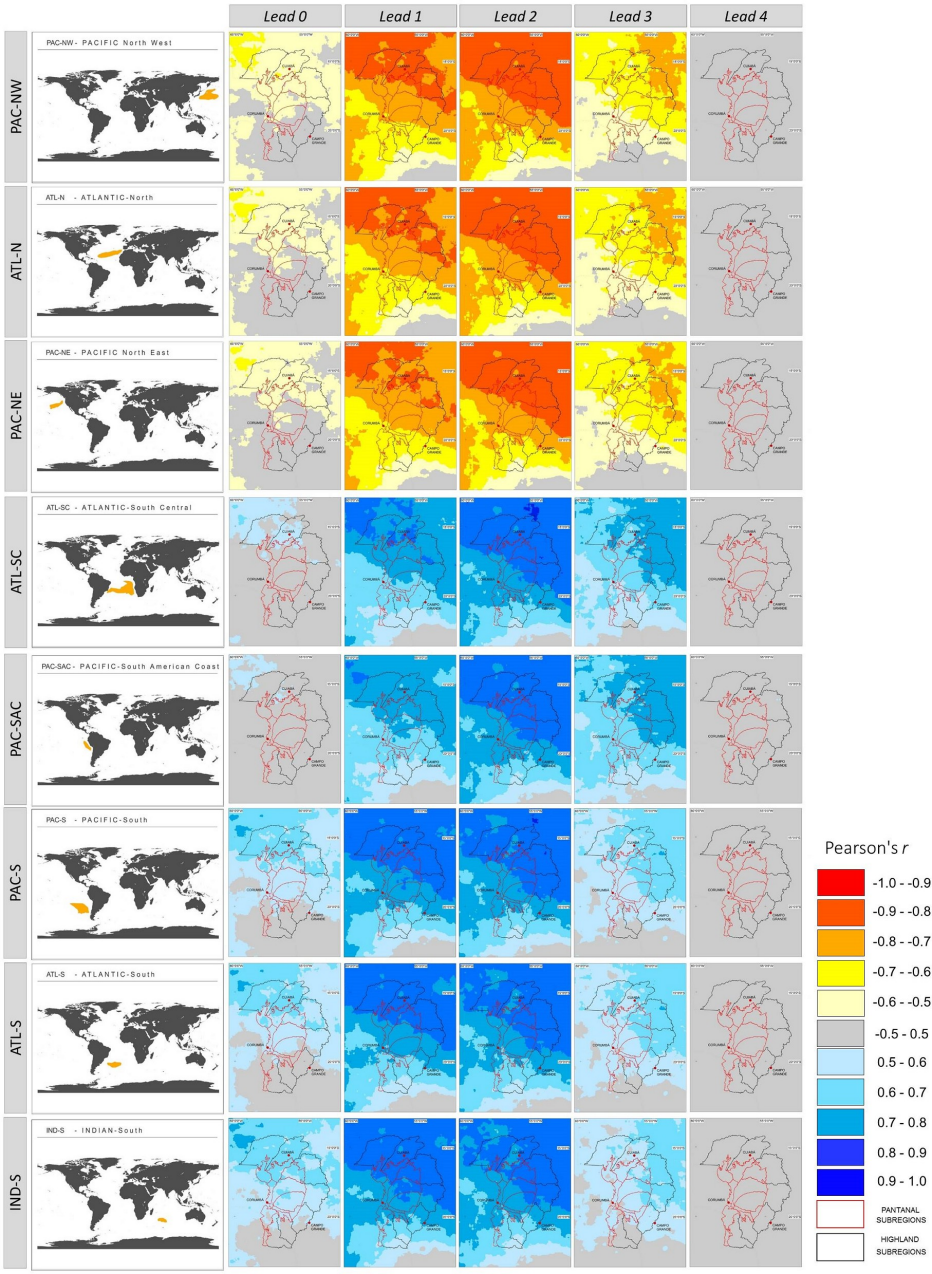
Spatial correlation (Pearson) between monthly mean precipitation for the Upper Paraguay River Basin (UPRB) and Sea Surface Temperature (SST) for different oceanic regions. Lead times are given in months (top) and oceanic regions are listed at left. Fig 7 shows location of oceanic regions.

Fig 8 shows the spatial correlation (Pearson) patterns between the oceanic SST and UPRB precipitation for different lead times. A lead time of two months from the maximum SST produced the highest correlation coefficients with precipitation. The coefficients were negative for PAC-NW, ATL-N, and PAC-NE and positive for ATL-SC, PAC-SAC, PAC-S, ATL-S, and IND-S. Fig 7 also indicates a northward gradient of increasing correlation values. This gradient suggests that northern sectors of the UPRB are more sensitive to higher SSTs in the oceanic regions considered in the present study.

**Fig 9:**
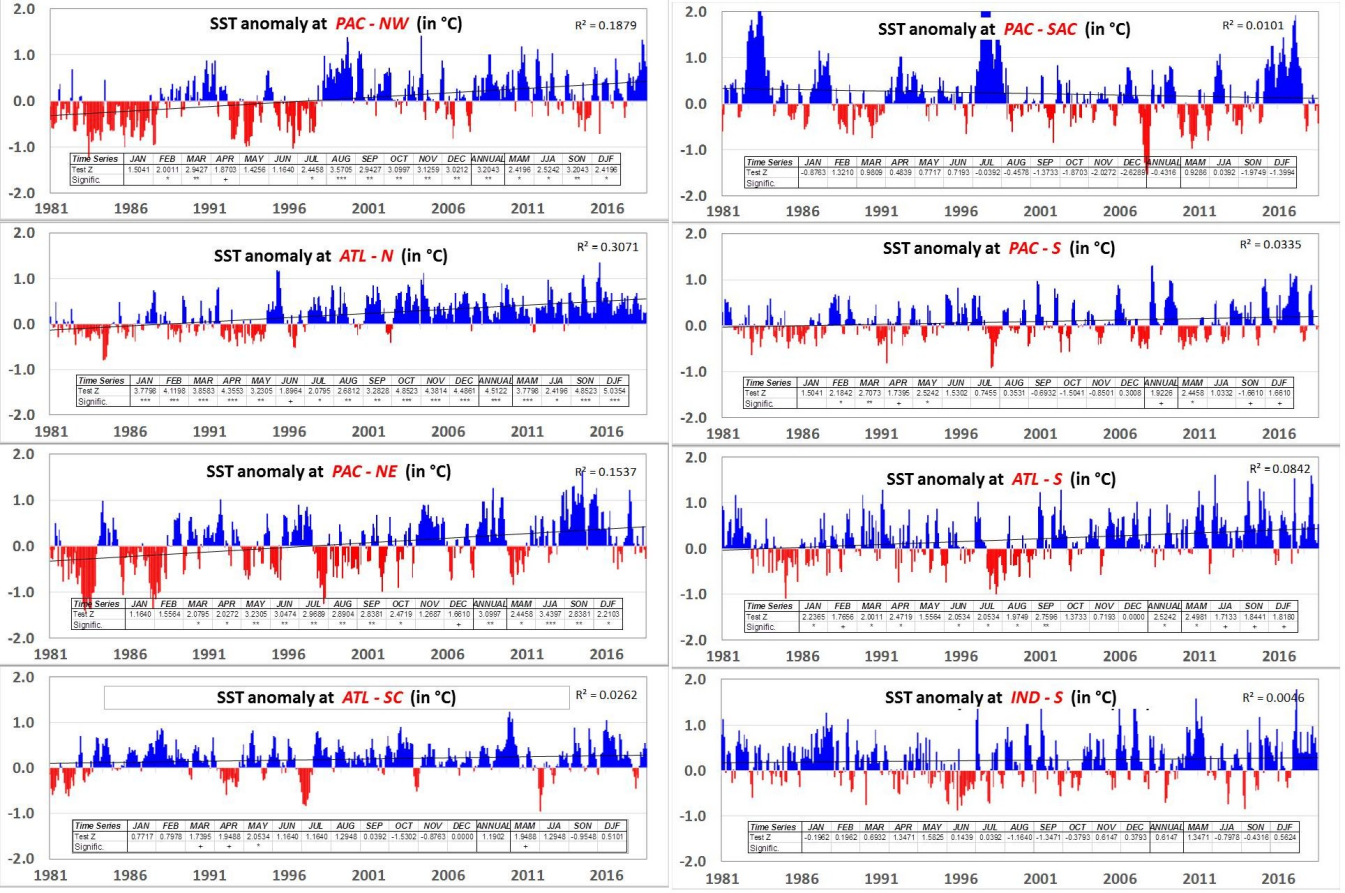
Anomaly trends in Sea Surface Temperature (SST) for different oceanic regions. Anomalies are in °C and based in the normal 1981–2010. Slope and trend significance test was performed with the Mann-Kendall method.

This research also identified trends in temporal anomaly dynamics (1981–2017) for the eight oceanic regions identified above. The PAC-NW and ATL-N showed a statistically significant (P < 0.001) increase in annual SST (Fig 9). The PAC-NE region also exhibited the same increase but with less statistical certainty (P < 0.01). As shown in Fig 7, the Northern Hemisphere oceanic regions showed a strong correlation with decreasing precipitation in the UPRB (Table 3, Fig 8). Of the Southern Hemisphere oceanic regions, only ATL-S showed a significant increase (P < 0.05) in annual SST. The increasing SST in this oceanic region implies an increase in UPRB precipitation.

## Discussion

In this study, CHIRPS v.2 proved to be a valuable tool monitoring extreme events and, as in studies made in Northeast of Brazil [45], it contributed to a better understanding of the spatial and temporal variability of monthly rainfall in the UPRB. Likewise, the application of the SPDI was most appropriate when interpreting temporal and spatial analysis of precipitation extremes in the UPRB.

The annual precipitation in the Highlands significantly exceeded that reported in the Pantanal. The analysis did not detect significant precipitation differences among the Highlands sub-regions but identified significant precipitation differences among the Pantanal sub-regions. The irregular spatial distribution of rainfall in the study area is somewhat attributable to regional relief, which causes orographic effects [14,46]. Pronounced relief act as obstacle to air flow capable of generating an orographic lifting and the development of convective precipitation affecting immediate areas [47]. As a result, a greater volume of rainfall occurs in the Highlands, thus entering the headwaters of the principal Pantanal rivers, i.e., the Miranda, Aquidauana, Taquari, São Lourenço, Paraguay, and Cuiabá [14].

Data from the 1981–2018 study period showed no significant differences in the annual precipitation distribution (i.e., loss of seasonality) among the major regions or sub-regions. Data analysis also failed to detect significant trends in annual precipitation in any particular region. However, two studies analyzing monthly trends in precipitation from 1977−2006 using 12 rain gauge stations in the Pantanal found a small decrease in precipitation associated with pronounced inter-annual variability [48,49]. The resulting variation might stem from differences in the time lapse considered as well as from limits related to the spatial data resolution. At a wider spatiotemporal scale, results from long-term trend studies of the past fifty years and projections of climatic models until the end of the 21st century, show no trend for the precipitations in the Amazon, in the Pantanal and in the Northeast of Brazil [50,18]. In the case of the Amazon basin, recent studies show evidence of an intensification of the hydrological cycle when considering shorter but more recent series [51,52].

The precipitation patterns exhibited considerable spatiotemporal heterogeneity in the study area. Overall, the SPDI data from the UPRB showed that 15.1% of the months experienced abnormal wet events. Of these, 90.6% of these wet events did not last longer than two months. The 1981–2018 data showed that 12.7% of the months experienced protracted dry events. The SPDI values of the Highlands were significantly different from those of the Pantanal.

The sub-regions of the Highlands, as well as those of the Pantanal, were grouped into three clusters. These clusters highlighted three extreme precipitation events, one wet and two dry, during the 1981–2018 study period. The events in question exerted varying effects on the different Highlands and Pantanal sub-regions. The Pantanal, particularly its central area, bore most of the effects of the extreme precipitation events. Information about spatiotemporal dynamics of such events is fundamental for wildlife management and nature conservation of the Pantanal. Plants and wild animals, for example, are affected by tree mortality in riparian forest after extreme flooding, with consequent habitat modification for wild animals [16]. Extreme hydrological events such as the ones described in the present study have been proven to impact the management of cattle raising in this region, preventing access to, circulation within, and occupation of some grazing areas [20].

Now, the seasonal cycle of precipitation over much of tropical South America, including the Pantanal, responds to the dynamics of the South America Monsoon System (SAMS) and the South Atlantic convergence zones (SACZ) [53,54]. Additionally, there is evidence that SAMS duration and amplitude obtained from the observed large-scale index have both increased in the last 32 years [55], as well as an increase of extreme precipitation and consecutive dry days have been observed in the SAMS region from 1969 to 2009 [56]. The IPCC [18] predicts a scenario of an increase of precipitation extremes and in the extension of monsoon area, while SAMS overall precipitation will remain unchanged. According to Bergier [54], these SAMS dynamic alterations have been generated by SST anomalies.

The SST from eight oceanic regions was strongly correlated with the UPRB monthly precipitation when modeled with a two-month offset (lead time). Other correlation patterns indicated that specific regions of the North Pacific and North Atlantic exerted strong negative effects on precipitation. Oceanic regions of the Southern Hemisphere (two Pacific, two Atlantic, and one Indian Ocean region) showed a significant positive correlation. Each of these oceanic regions showed a correlation between the SST and UPRB precipitation that increased in a northward direction. The results from the present study show a higher resolution than do those of Batista Silva et al. [57], regarding which oceanic regions’ SSTs correlate best with the UPRB precipitation as well as with the lead time. This difference may be referred to hidden effects from spatial heterogeneity in soil moisture and natural water storage in river flow dynamics are still not well represented on climate models, limiting the prediction capability of hydrologic extremes [9].

The three Northern Hemisphere oceanic regions that correlated negatively with the UPRB precipitation also exhibited significant increases in their SSTs. Although there is a ‘recent’ warming (since the 1950s) at all latitudes in SST over each ocean, the warming is more prominent in the Northern Hemisphere, specifically in North Atlantic, showing a positive trend in SST since the 1980s which is consistent with reversing trends in the Walker Circulation [18].

Such warming trend mirrored an increase in extreme dry events for the UPRB that was expressed by more intense and/or prolonged dry spells. Models predict a 10–20% reduction in rainfall in the Pantanal over the next 20 years and of 30% from 2071 on [8]. According to Keenlyside et al. [58], this warming trend may last for the next few decades, in response to both present day atmospheric concentrations of greenhouse gases and projected future changes in radiative forcing. Drought projections should be reevaluated in terms of the newly identified causal connections between the Pantanal dry pulses and the increasing Northern Hemisphere SST. This adjustment would allow us to determine whether predicted conditions are consistent and supported by current data and models or whether these conditions indicate larger scales and more complex climate dynamics.

Studies of the North Atlantic SST [57,59] have shown that large, inverse correlation effects (coefficients greater than 0.6) occur north of 50°N. This study detected even higher correlation coefficients (r = -0.88) for the North Atlantic oceanic region ATL-N. As part of SAMS dynamics, tongues of vapor emerge from Amazonia in summer transporting essential rainfall to southerly regions comprising, among others, that of the UPRB [60]. The teleconnections between positive anomalies of North Atlantic and lower than average precipitations in the Pantanal is possibly due to alterations to the intensity and position of the South America Low-Level Jet (SALLJ) streams affecting the southward transport of rainforest evapotranspiration moisture from the Amazon basin to the Pantanal [54,61]. Further studies are needed to better describe the effects of sea-air interactions on river levels in Pantanal and Amazonia [61].

Some research has suggested that the SST resulting from Atlantic El Niño events can explain the inter-annual variation in precipitation for some regions of South America [59]. The easterly region ATL-SC considered in this study encompasses oceanic areas in which the Atlantic Niño develops (ATL1-ATL3). The data presented here indicate that the phenomena explain over 80% of the precipitation variability observed for the UPRB, a value that is much higher than the 50% obtained by Batista Silva et al. [57]. The westerly section of the ATL-SC as well as the ATL-S [62,63] exhibited SST values that were strongly correlated with enhanced precipitation in the South Atlantic convergence zone (SACZ), which in turn, affects the UPRB´s inter- and intra-annual precipitation dynamics. The lack of significant correlation trends from the Southern Hemisphere oceanic SST suggests that they will not cause significant variations in the frequency of UPRB extreme wet events outside those that were observed for the 1981–2018 time series analyzed here.

For the Pacific, there is an evident linear correlation between the SST of a large part of the Pacific basin and the Pantanal precipitation dynamics. Similar to the results from Batista Silva et al. [57], correlation is negative with North Pacific SST and higher absolute values than those located over the South Pacific at the same latitude belts. Even though no direct relation could be established between flood peaks in the Pantanal and ENSO events [64], the SST from the NIÑO1+2 region (0–10°S, 90–80°W) showed a positive correlation strong enough to affect the Pantanal precipitation [11]. The PAC-SAC region analyzed by our study corresponds to the NIÑO1+2 index.

As for the impact of Indian Ocean SST positive anomalies on South American precipitation (IND-S, in the present study), it seems to be the result of a combined response to El Niño events, via alterations of the Walker circulation pattern and through a mid-latitude wave-train teleconnection [65]. The Indian Ocean Dipole IOD [66], measured as the difference in SSTs between the Arabian Sea (western pole) and the eastern Indian Ocean south of Indonesia (eastern pole), play an important role in precipitation variability in South America [67]. Positive phase of ENSO is associated to Positive phase of the Indian Dipole, which could trigger wave trains to South America. Behera and Yamagata [68] showed that IOD modulates the Darwin pressure variability, that is, one pole of the Southern Oscillation and could influence ENSO [69,70]. The correlations between precipitations at the UPRB with more than one oceanic region stresses the importance of future studies evaluating combined influence.

Besides ENSO, the existence of important interconnections between hydrological dynamics and oceanic SST variability through atmospheric circulation of moisture from oceanic sources have also been confirmed for other important macroclimate indexes such as PDO [71,72]. Similar to the PAC-SAC-ENSO relations, PAC-NW and PAC-NE SST anomalies covary closely with UPRB precipitation on decadal time scales due to variation introduced by major climate oscillations such as the PDO. The time scale of the PDO is around 20 to 30 years, and relative to the presence of positive or negative SST anomalies along the coast of western North America; it comprises PAC-NW, the oceanic region from this study with the strongest and negative correlation to UPRB precipitation dynamics. PDO has the largest capability to modulate the climate in the UPRB [27,57]. The current phase of the PDO is positive, and consequently, anomalous warm at PAC-NW. According to IPCC´s last Assessment Report (AR5), the confidence is low in projections of future changes in PDO phase [18].

Predictability is seen to be high in tropical regions of South America where the models respond well to the SST forcing, where high skills have been reached for Northeast Brazil [73]. The correlations between precipitations at the UPRB with more than one oceanic region stresses the importance of future studies evaluating combined influence.

## Conclusion

1. The present study predicts severe droughts for the Pantanal ecosystem, and these droughts will be triggered by a warming of SSTs occurring in the Northern Hemisphere (North Atlantic and North Pacific oceans, Figs 7 and 9).
2. The predictability of extreme precipitation and drought events was improved by a resulting two-month reduction of the lead-time between the SST warming and the concomitant precipitation impacts, and this new lag time explained 80% of the Pantanal´s precipitation variation from specific oceanic regions, comprised in the major oceanic indexes (e.g., ENSO, PDO, NAO, ATL3).
3. These climate change impacts will result in changes in the inter- and intra-annual flooding dynamics, drastically affecting the functioning of the Pantanal ecosystem, with consequences for wildlife diversity and distribution, as well as for the sustainability of ongoing human activity that relies on these wetland ecosystem services.
4. Regarding foreseeable global climate and land use change scenarios, the results from the present study provide solid evidence that can be used by different decision-making levels (from local to global) in the identification of the most appropriate management practices and for effectively achieving sustainability of the anthropic activity occurring in the Pantanal.

The results from present study exhaustively attended a well identified need to assertively categorize, at a local scale, responses from global change dynamics [7,31,50,74–79], that is, the quantification of the effect on Pantanal´s precipitations generated by significant global changes such as SST variations from the different specific oceanic regions. Nevertheless, there is still an additional need to discern and quantify from the Pantanal's hydrological functioning and balance how much corresponds specifically to the effects of an on ongoing intensification of local anthropogenic activities.

To cope with this need, we are currently conducting studies analyzing the SST and precipitation trends for an extended period (e.g., 1951 to present), as well as quantifying coeval local land cover and land use changes, and the concomitant variability in inter- and intra-annual Pantanal´s flooding. The integration of such data in a multiscale model will allow to discern the various determinants acting at different space and time scales, capable of severely affecting Pantanal´s ecosystem functioning and integrity. Thus, identifying the most assertive management practices oriented to mitigate the effects of the most important global changes scenarios, ultimately aimed for the conservation of the largest wetland in the world and one of the most important biodiversity hotspots.
